# Cases of Diseased Placenta

**Published:** 1854-04

**Authors:** E. C. Atkinson

**Affiliations:** Dover, Lee Co.


### *Cases of Diseased Placenta and Umbilical Cord.*

REPORTED BY E. C. ATKINSON, M. D., DOVER, LEE CO.

On May 1853, I was summoned to attend Mrs. F. during her fourth parturition at full time, a lady of good health, good habits, and free from constitutional taint of any kind. Her periods of gestation have always previously been congenial. About three weeks previous to her present parturation however she began to experience a peculiar nerveless languid feeling acompanied with headache loss of appetite, and gloomy apprehensions, for which she had sought no advice. At this time her countenance exhibited a dusky jaundiced hue for which I could discover no tangible cause. Her labor was natural and easy. Near the close of it the membranes were ruptured, and the usual quantity of liquor amnii escaped being mixed with a sediment which gave it a brown colour, and very offensive smell. On attempting to tie the umbilical cord after delivery I was surprised to find the ligature to cut it in two, and on removing the child and secundines for inspection, the following phenomena were presented. The fœtal extremity of the cord was of a dark brown color, which gradually became black, as it approached the placenta, about one half the natnral size, and very soft being readily crushed by slight pressure between the thumb and finger. At the other extremity it was still more diseased being ruptured by the sligtest traction and had been separated from the placenta during labor. The centre of the free surface of the placenta exhibited a semiputrid appearence over a space of about three and a half inches in diameter, surrounding the funis which was gradually lost in healthy tissue as it approached the border. The attached surface appeared healthy except a brown spot in the centre, which showed that the disease had extended quite through the placental mass.

The skin of the child was of a deep yellow color over the the entire body. The child was emaciated and sluggish, and exhibted symptoms of extreme languor and debility. These however were gradually relieved by the use of gentle friction and warm baths. At the end of fifteen days when the yellowness of its surface had mostly disappeared, it become covered with dark brown blotches of different sizes giving it a most singular maculated appearance. These soon disapper-ed and it continued to improve untill about two months old when it suffered from an attack of erysipelas. It however recovered and has since been healthy. The mother also recovered without difficulty. It seems evidently in this case that the diseased condition of the child at birth, and the momorbi feelings of the mother prior to parturition were cause by putrid matter from the diseased tissues circulating in the blood, and it is highely probable that the disorganising of those tissues could not have advanced much farther without the child being destroyed by a rupture of the cord, and the life of the parent at least endangered, by the resulting hemorrhage.

CASE 2. In the month of June 1853, I was called to see Ms. W. a lady forty years of age and of good health at this time, though in former years she suffered a great deal from uterine disease, when I arrived I found that miscarreage at about the ninetieth day of pregnancy had taken place I found the foetus in a deseased state much smaller than natural, and the umbilical cord larger seane expulsory pains were at this time going on. I gave her a few grains of ergot and left. On returning a few hours after I found the placenta had been expelled, which presented the following appearence. Size about that of larger walunt pearshaped and this shape apparently moulded by the cavity of the uterus, half of which it must have filled. About half an inch of the uterine end looked healthy, the remainder was highly vascular harder than natural, and presented the general appearance of fungoid groath. From as acurate an examination of it as I could make without the aid of a microscope, I could pronounce it nothing else than a fungus growth of the plsaenta. The patient informed me that for the past four weeks, she had had almost constant pains in the uterine region which gradually increased untill the occurrence of abortion.

